# Evaluation of the Effectiveness of an Innovative Polycomponent Formulation on Adult and Aged Human Dermal Fibroblasts

**DOI:** 10.3390/biomedicines11092410

**Published:** 2023-08-28

**Authors:** Francesca Rosaria Augello, Francesca Lombardi, Serena Artone, Alessia Ciafarone, Serena Altamura, Luisa Di Marzio, Maria Grazia Cifone, Paola Palumbo, Maurizio Giuliani, Benedetta Cinque

**Affiliations:** 1 Department of Life, Health & Environmental Sciences, University of L’Aquila, 67100 L’Aquila, Italy; francescarosaria.augello@univaq.it (F.R.A.); maurizio.giuliani@univaq.it (M.G.); 2 PhD School in Medicine and Public Health, Department of Life, Health and Environmental Sciences, University of L’Aquila, 67100 L’Aquila, Italy; 3 PhD School in Health & Environmental Sciences, Department of Life, Health and Environmental Sciences, University of L’Aquila, 67100 L’Aquila, Italy; 4 Department of Pharmacy, University of Chieti—Pescara “G. D’Annunzio”, 66100 Chieti, Italy; 5 Unit of Plastic and Reconstructive Surgery, Casa Di Cura Di Lorenzo SrL, Via Vittorio Veneto 37, 67051 Avezzano, Italy

**Keywords:** polycomponent formulation, skin aging, dermal fibroblast, human recombinant polypeptide of collagen-1 α chain, TGF-β1, oxidative stress

## Abstract

Skin aging is a dynamic process that determines structural alterations in ECM and reduction in dermal fibroblasts. The recent availability on the market of an innovative polycomponent formulation (KARISMA Rh Collagen^®^ FACE, K) containing noncrosslinked high-molecular-weight hyaluronic acid (HMW-HA), a human recombinant polypeptide of collagen-1 alpha chain, and carboxymethyl cellulose (CMC), attracted our scientific interest in evaluating its biomolecular effects on human dermal adult and aged fibroblasts. After treatment with increasing K concentrations, cell proliferation, collagen I, prolyl 4-hydroxylase (P4HA1), an essential protein in collagen biosynthesis, and α-SMA levels were assessed. The fibroblast contractility, TGF-β1 levels, and oxidative stress markers were also evaluated. K formulation exposure led to a significant and dose-dependent increase in the proliferation and migration of adult fibroblasts. Of note, the K exposure counteracted the H_2_O_2_-induced aging by promoting cell proliferation, reducing β-galactosidase activity, and neutralizing the aging-associated oxidative damage. Moreover, an increase in collagen I, P4HA1, α-SMA, TGF-β1 levels, and improved contractility of adult and aged fibroblasts were observed after treatment. Overall, our results show evidence that the K treatment is efficacious in improving biological functions in adult fibroblasts and suppressing the biomolecular events associated with H_2_O_2_-induced cellular aging, thus supporting the regenerative and bio-revitalizing action of the K formulation helpful in preventing or treating skin aging.

## 1. Introduction

Skin structure and physiology are continuously subjected to intrinsic and extrinsic factors which gradually damage the skin homeostasis, inducing a progressive loss of tissue integrity and impairment of cellular functions. At the tissue level, the epidermis becomes thinner due to a progressive slowing of keratinocyte proliferation and differentiation failing. Of note, the stimulation of the regenerative capacity of keratinocytes is a potential target in antiaging therapy [1,2]. Regarding the dermal compartment, the cellular and molecular mechanisms underlying skin aging are associated with the dermal fibroblasts in terms of number and functionality and with the alteration of ECM homeostasis, involving mainly collagen, the crucial fiber-forming structural element [3]. In the aged skin, the number of dermal fibroblasts functionally active and responsible for the synthesis of the ECM elements is reduced while an accumulation of aged fibroblasts occurs. These cells are characterized by altered metabolic functions with reduced collagen synthesis in favor of increased secretion of inflammatory cytokines, chemokines, and matrix metalloproteinases (MMPs), degradative enzymes of ECM components, such as collagen fibrils [4]. All these characteristic features significantly reduce the skin’s regenerative potential [5]. In the process of aging, the skin structure and integrity are impaired with changes in mechanical properties and viscoelasticity due to the exhaustion of the ECM components, such as collagen, elastin, and hyaluronic acid (HA) [6]. Then, during the aging process, the ECM, which generally serves as the physical support structure for the dermal connective tissue and makes up the appropriate environment for active and functional dermal cells, is altered and impacts the functions of the fibroblasts that cannot adhere to it, being the mechanical forces reduced. The aged dermal fibroblasts appear with collapsed cytoplasm, rounded shape, and, in particular, a reduced capacity of type I collagen production [7].

For the above, dermal fibroblasts represent one of the most exciting targets of skin aging prevention or therapy.

To hinder or counteract the critical signs of skin aging, the injectable hydrogel is one of the most used approaches due to peculiar physicochemical characteristics. The known clinical efficacy attracted our scientific interest in evaluating deeper the biological effects on in vitro cellular models. This innovative commercially available polycomponent formulation (KARISMA Rh Collagen^®^ FACE, K) includes three components, generally used individually. In particular, K formulation contains, in well-defined proportions, noncrosslinked high-molecular-weight HA (HMW-HA), recombinant polypeptide of collagen-1 chain, and carboxymethyl cellulose (CMC). HA is an essential constituent of the extracellular matrix with multiple biological activities, including hydration, that contribute to the firmness and bounciness of the skin. Its hydrophilic properties draw water, endowing improved viscoelasticity to the skin. The literature data extensively report that the hydrogels containing HA are able to improve skin appearance and texture [8,9]. Clinical observations demonstrate that the positive effects of injectable hydrogels can be persistent, going beyond the filling impact [10], suggesting that other mechanisms could be involved to stimulate ex novo collagen synthesis and generally improve the functionality of fibroblasts. The innovative polycomponent K formulation includes the recombinant polypeptide α1 chain of type I human collagen synthesized by transgenic silkworms. Due to its chemical structure, the recombinant α1 chain cannot form the collagen triple helix [11]. When used at concentrations > 5 µg/mL, it promoted the adhesivity and spread of human dermal fibroblasts (HDFs) similarly to gelatin or native collagen. The results suggested the advantageous use of recombinant α1 chain of collagen as biomaterial for a variety of medical applications, having a low risk of bacterial contaminations and a quality of product easily controlled [11]. It is important to highlight that the collagen for injectable formulations is generally extracted from different animal sources [12,13], and that the human collagen formulations are also available as a valid alternative. However, some pitfalls can be found, such as long preparation times, high costs for the procedure, and highly skilled teams, so an exciting alternative is represented by recombinant collagens [14]. Carboxymethylcellulose (CMC) is an FDA approved water-soluble polysaccharide, derived from cellulose [15]. Of note, CMC tends to disperse in water to form a transparent colloidal solution, i.e., CMC gel, and it is also able to suspend solids in aqueous media and stabilize emulsions. It is also used as an agent to thicken, bind, and gel and to retain water [16]. The CMC has been used in the biomedical field due to its biocompatibility, low cost, nontoxicity, good biodegradability, and low immunogenicity. The numerous rheological properties of CMC such as mechanical strength, different formability, tunable hydrophilicity, and viscosity justify a wide range of applications [17] rendering the CMC the material of choice in injectable formulations [15,18]. Among CMC-based hybrids, the hydrogel has gained much interest also in pharmaceutical and cosmetic applications, since the CMC ensures the recommended rheological properties due to its viscosity. In soft tissue engineering, the CMC hydrogels offer a softer feel via high spreading accessibility improving the dermal formulation administration and making it less viscous [19].

Herein, the effects of K formulation treatment at different concentrations and for different incubation times were evaluated on proliferation, migration, and contraction of adult and aged HDFs. The influence of K formulation exposure on type I collagen synthesis pathway was also assessed. Considering the key role of TGF-β1 pathway on collagen I synthesis, the modulation of its levels was also investigated in both adult and aged dermal fibroblasts after treatment with K formulation. Furthermore, the ability of K formulation to counteract oxidative stress associated with H_2_O_2_-induced aging process in fibroblasts was verified. Our findings support the efficacy of K formulation as a promising strategy for maintaining dermal fibroblast homeostasis and inhibiting critical age-related skin signs.

## 2. Materials and Methods

### 2.1. Preparation of K Polycomponent Formulation for Cell Treatments

KARISMA Rh Collagen^®^ FACE, a bio-restorative formulation (following named K) (kindly provided by Taumedika Srl, Rome, Italy), includes 200 mg/mL high-molecular-weight hyaluronic acid (HA), 200 µg/mL human recombinant collagen α1 chain, and 40 mg/mL carboxymethylcellulose (CMC). For cell treatments, K was prepared using the “extraction dilution method” as described by the UNI EN ISO 10993 regulation [20]. Briefly, the procedure was carried out in an extraction medium consisting of DMEM, supplemented with 10% of fetal bovine serum (FBS), 100 U/mL penicillin, 100 mg/mL streptomycin, and 2 mM glutamine (complete medium) at 37 °C ± 1 for 24 h, by continuous agitation. Subsequently, K was serially diluted and used at different concentrations (range: 0.1–10% *v/v*, final concentration).

### 2.2. Cell Systems

Adult normal primary human dermal fibroblast (NHDFs, Adult CC-2511) cell line, derived from a 47-year-old Caucasian female, was obtained from Lonza/Cell Applications (Basel, Switzerland) and cultured at low passage (N = 6–9) in complete medium in a humidified atmosphere of 95% air and 5% CO_2_ at 37 °C. All reagents and consumables were purchased from EuroClone (West York, UK). At the 15th passage the cells were used for the experiments.

To induce the aged phenotype, NHDFs at 70% confluence were treated with 25 μM hydrogen peroxide (H_2_O_2_) in PBS for 1 h. Then, the H_2_O_2_ solution was replaced with the complete medium. After 24 h, the cells (aged HDFs) were treated with K formulation at the different concentrations.

### 2.3. Treatments and Assessment of Cell Viability and Growth Rate

Cell growth rate was measured by IncuCyte^®^ Live Cell Imager system (Essen BioSciences, Inc., Ann Arbor, MI, USA) for real-time analysis of cell confluence. Briefly, adult or aged HDFs were seeded on a 96-well culture plate at 2.5 × 10^3^ cells/well and allowed to attach overnight. After, the cells were incubated with increasing concentrations (0.1, 0.5, 2, 5, or 10% vol/vol, final concentration) of K. Each treatment condition was replicated in three different wells, and three independent experiments were carried out. Culture plates were placed into IncuCyte^®^ instrument, and images were acquired every 4 h from 0 to 72 h after treatment. Two pictures were obtained from several points of the well, at 10× magnification. To measure the proliferation rate, cell confluence was analyzed by IncuCyte ZOOM™ software (2020b, Essen Bioscience, Newark, UK).

To analyze their number and viability, adult or aged HDFs were seeded at a density of 7000 cells/cm^2^, grown for 18 h, and incubated with K at 0.1–10% final concentration for 72 h in complete medium. Then, the cells were collected, centrifuged for 10 min at 400× *g*, and counted with 0.04% trypan blue (EuroClone, West York, UK), using a Bürker chamber, and visualized with Eclipse 50i microscopy (Nikon, Tokyo, Japan). Untreated cells were used as controls. For all the other experiments, similar cell conditions were used.

### 2.4. Staining Cells for β-Galactosidase Activity

*β*-Galactosidase activity, a indices of dermal fibroblast cellular aging in vitro, was assayed using a kit by Cell Signaling Technology (Danvers, MA, USA) according to the manufacturer’s instructions. After treatments, the aged HDFs were washed with cold PBS and fixed for 15 min with 1 mL of fixing buffer at room temperature. After washing, the cells were incubated with 1 mL of β-galactosidase staining solution containing 5-bromo-4-chloro-3-indolyl-β-d-galactopyranoside (X-gal) for 24 h at 37 °C. Images were obtained using a light microscope (Eclipse 50i, Nikon, Tokyo, Japan). Ten random fields were counted to determine the percentage of β-galactosidase-positive cells in the total cell population.

### 2.5. Fluorometric Measurement of Reactive Oxygen Species (ROS) with DCFH-DA

Intracellular ROS production was evaluated using a DCFH-DA probe (Immunological Sciences, Rome, Italy). The DCFH-DA probe is cell permeable and is subjected to deacetylation by esterase to produce nonfluorescent DCFH, which is retained in the cytosol. In the presence of ROS, DCFH is oxidized to fluorochrome 2′,7′-dichlorofluorescein. Thus, this probe has been utilized as an indicator of oxidative stress in biological systems. Briefly, after treatments, the aged HDFs were incubated with DCFH-DA solution (25 µM) at 37 °C for 30 min. ROS levels were then analyzed using VICTORX4™ fluorometer (PerkinElmer, Waltham, MA, USA) with excitation and emission filters of 488 and 535 nm, respectively. The values obtained were normalized for cell number and expressed as relative fluorescence unit (RFU)/10^5^ cells.

### 2.6. Malondialdehyde (MDA) ELISA Kit

The aged HDFs supernatants, recovered following treatment with K at 0.5, 2, or 5% up to 72 h, were assayed for malondialdehyde (MDA) levels by an enzyme-linked immunosorbent assay (ELISA) kit (Elabscience, Houston, TX, USA) as described in the manufacturer’s instructions. The values obtained were normalized for cell number and expressed as ng/10^5^ cells.

### 2.7. Western Blot Analysis

Western blot was used for protein detection. Cell pellets were washed in PBS and then lysed in RIPA Lysis Buffer (Merck KGaA, Darmstadt, Germany) added with 100 mM protease inhibitor cocktail (Sigma-Aldrich, St. Louis, MO, USA). Then, to eliminate cell debris, the samples were centrifuged at 17,949× *g*, and the total protein concentration was determined by DC Protein Assay (BioRad, Hercules, CA, USA). A total of 25 μg of proteins was separated by 10% SDS-polyacrylamide gel electrophoresis and transferred onto 0.45 µm nitrocellulose membrane sheets (BioRad) for 1 h at 4 °C. We used 5% nonfat dry milk to block the aspecific sites on membranes (one hour at room temperature). The membranes were then incubated overnight at 4 °C with rabbit polyclonal antibody anti-COL1A1 (Boster Biological Technology, Pleasanton, CA, USA) 1:1000, rabbit monoclonal antibody anti-P4HA1 (OriGene, Rockville, MA, USA) 1:1000, or mouse monoclonal antibody anti-α-actin smooth muscle (ACTA2, α-SMA) (OriGene, Rockville, MA, USA) 1:1000 and mouse monoclonal antibody anti-GAPDH (OriGene, Rockville, MA, USA) 1:1000. Horseradish peroxidase (HRP)-conjugated goat anti-rabbit or rabbit anti-mouse IgG secondary antibodies at 1:2000 were used (Millipore EMD, Darmstadt, Germany). The densities of immunoreactive bands visualized by chemiluminescence reagent (ECL, Amersham Pharmacia Biotech, Buckinghamshire, UK) were quantified by chemiluminescence documentation system ALLIANCE (UVITEC, Cambridge, UK). The data were normalized to the relative GAPDH bands.

### 2.8. Immunofluorescence Assay for Type I Collagen

Adult or aged HDFs were grown on coverslips of a 12-well plate (seeded at 7000 cells/cm^2^) and untreated or treated with K at 5% for 72 h. At the end of treatment, the cells on the coverslips, after washing with PBS and formaldehyde fixation (4% for 20 min), were permeabilized with 0.1% Triton X-100 (Sigma-Aldrich, St. Louis, MO, USA) for 5 min and blocked with 3% BSA (Sigma-Aldrich) for 20 min at room temperature. After that, coverslips were incubated for 18 h at 4 °C with rabbit polyclonal antibody anti-COL1A1 (Boster Biological Technology, Pleasanton, CA, USA) at dilution 1:250. FITC conjugated goat anti-rabbit polyclonal IgG secondary antibody (Millipore EMD, Darmstadt, Germany) was used at 1:1000 for 1 h at room temperature. VECTASHIELD^®^ Antifade Mounting Medium with added DAPI (Vector Laboratories, Inc., Burlingame, CA, USA) was used to mount the coverslips examined at 100× magnifications by fluorescence microscopy (Eclipse 50i, Nikon, Tokyo, Japan).

### 2.9. Extracellular Collagen Quantification

The extracellular collagen from cell culture supernatants was quantified using the human type I collagen ELISA kit (Immunological Sciences, Rome, Italy). Adult and aged HDFs were grown on a 12-well plate (seeded at 7000 cells/cm^2^) and treated with K at 0.5, 2, or 5% for 72 h in a complete medium. Following the K exposure, the media were recovered and centrifuged at 1000× *g* for 15 min to remove the cellular debris/dead cells. The concentration of collagen I was assayed, and the obtained values were normalized for cell number and expressed as ng/10^5^ cells.

### 2.10. In Vitro Scratch Assay

The effect of K formulation on migration/proliferation in adult HDFs was carried out by scratch assay [21]. The cells were seeded at 20 × 10^4^/cm^2^ on multiwell plates and left to grow until reaching confluence. In the absence of the medium, the cell monolayers were scratched with a 200 µL pipet tip and then washed with PBS to remove debris. Fresh medium, containing or not K at 0.5, 2, or 5%, was added to the scratched monolayers. The images were acquired by the inverted light microscope (Eclipse TS100, Nikon, Tokyo, Japan) at different time points after the scratch (0–24 h). For the quantitative analysis, the standalone TScratch software was used. The software was able to measure the portion of the area that was occupied by the cells by a mathematical model and then to calculate the percentage of wound closure. The experiments were performed in duplicate, and six fields for each condition were evaluated.

### 2.11. Collagen Gel Retraction Assay

To evaluate the contraction ability of adult or aged HDFs, the fibroblasts were put into three-dimensional collagen lattices as previously described [22]. Briefly, acid-extracted type I collagen (5 mg/mL) mixture was made on ice with rat tail collagen I (Enzo Life Sciences, Lausen, Switzerland) in acetic acid 0.2%. The solution was then diluted at 3 mg/mL in sterile water, and the cells were resuspended in complete media (8 × 10^5^ cells/mL).

Resuspended cells (8 × 10^4^/100 µL) were mixed to 300 µL complete media, 200 µL collagen mixture (3 mg/mL), and the mixture was quickly neutralized with NaOH on ice. These treatments did not influence the viability and the number of fibroblasts, as revealed by Trypan blue exclusion test (not shown). A total of 500 µL of the collagen/cell mixture was placed into each well of a pre-warmed 12-well plate (Corning Incorporated, Corning, NY, USA). After gel polymerization (at 37 °C for 1 h), the media containing or not K (0.5, 2, or 5%) were added in each well. Then, the lattices were accurately detached from the well. The images of lattices were taken with a digital camera before release and multiple times after release. The lattice area was evaluated by ImageJ and normalized to pre-release area (T0).

### 2.12. TGF-β1 ELISA

TGF-β1 levels were assayed in the cell supernatants by human TGF-β1 ELISA kit (Sigma-Aldrich, Saint Louis, MO, USA), as reported in the manufacturer’s instructions. The adult or aged HDFs were plated at 7000 cells/cm^2^ and exposed to K at 0.5, 2, or 5% up to 72 h. Next, the media were centrifuged at 1000× *g* for 15 min to clarify them from cellular debris/dead cells. The TGF-β1 concentration was then measured by ELISA kit. The obtained values were normalized for cell number and expressed as pg/10^5^ cells.

### 2.13. Statistical Analysis

All data were evaluated by GraphPad Prism version 6.01 (GraphPad Software, San Diego, CA, USA). To compare the mean values among groups, one-way ANOVA or two-way ANOVA, followed by Dunnett’s post hoc test, was used. Data were expressed as mean ± SD or mean ± SEM as reported in figure legends. The *p* values were considered statistically significant when lowered than 0.05.

## 3. Results

### 3.1. Effect of K Formulation on Cell Proliferation of Human Adult and Aged Dermal Fibroblasts

**Figure 1:**
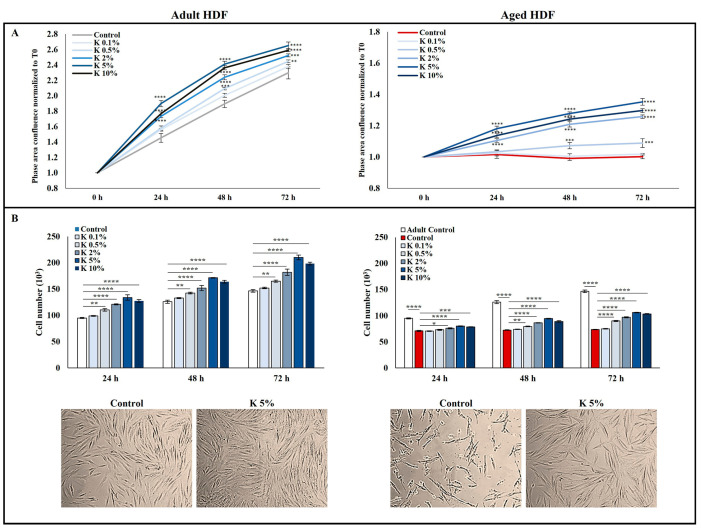
Effect of K formulation on proliferation rate and cell number of adult and aged HDFs. (**A**) Growth curves were analyzed as cell confluence through IncuCyte^®^ instrument and monitored up to 72 h. Adult and aged HDFs were treated with K formulation at different concentrations (0.1, 0.5, 2, 5, and 10%) in complete medium. Data from one representative out of three independent experiments are reported as mean ± SD. (**B**) The cell number of adult and aged HDFs, treated as described in (**A**) and stained with trypan blue, was evaluated. Data obtained from three experiments, in duplicate, are expressed as mean ±SEM. Representative phase-contrast microscopic images of adult and aged HDFs treated for 72 h with K (5%) are shown (10× magnification). The comparative analysis of groups of data was performed by the two-way analysis of variance (ANOVA) followed by Dunnett’s post hoc test (* *p* < 0.05, ** *p* < 0.01, *** *p* < 0.001, **** *p* < 0.0001).

To evaluate the biological effects of K formulation, we used two different cultured fibroblast models. First, a normal human adult fibroblast cell line, derived from a 47-year-old female subject, was used at low passage (N = 6–9) to recapitulate the behavior of normal and adult HDFs and treated with different concentrations of K (0.1, 0.5, 2, 5, and 10%, *v/v*) for 24, 48, and 72 h. The second used model consisted of HDFs treated with H_2_O_2_ 25 µM for one hour according to the one-step model [23] to obtain aged HDFs and then exposed to K. As shown in Figure 1A, the two models stood out significantly in the proliferation rate, evaluated by dynamically monitoring up to 72 h and expressed as cell confluence. The adult HDFs (controls) showed a gradual growth, while in the aged fibroblast model, the cell confluence, as expected, did not increase remaining constant over time. The effect of different concentrations (0.1, 0.5, 2, 5, and 10%, *v/v*) of K formulation on adult and aged HDFs was first evaluated with respect to cell proliferation. As shown in Figure 1A, the proliferation rate of the adult HDFs exposed to increasing concentrations of K augmented more quickly than that of untreated HDFs, and the rise was time- and dose-dependent, being statistically significant starting from 0.5%. Interestingly, when the aged HDFs were exposed to K, the cell confluence enhanced in a time- and concentration-dependent manner compared to that of the relative untreated controls; the 5% concentration was the most effective in stimulating the proliferation of aged HDFs. The analysis of the K formulation effect on the cell number showed a similar trend in both models (Figure 1B). The cell number of the adult HDFs was always significantly greater than aged HDFs. The exposure of the adult and aged HDFs to K formulation caused a marked increase in the cell number compared to that in the relative controls as incubation time and concentrations increased. Notably, 5% K was the most effective concentration in inducing an increase in the cell number compared to the untreated cells in both the adult and aged HDFs (Figure 1B). Representative images from microscopic observations of adult and aged HDFs treated with 5% K for 72 h confirmed the increase in the cell number. The aged HDFs appeared shrunken and morphologically different from the adult HDFs. After the treatment with 5% K, the aged HDF morphology returned to that more like of the adult HDFs (Figure 1B). This result indicates that K formulation could counteract the effects of aging on the proliferation rate as well as cell morphology. Based on the obtained results, the concentrations of K at 0.5, 2, and 5%, being the most efficacious in stimulating HDF proliferation, were chosen for the following experiments.

### 3.2. Effect of K Formulation on β-Galactosidase Activity and Oxidative Stress in Aged HDFs

**Figure 2:**
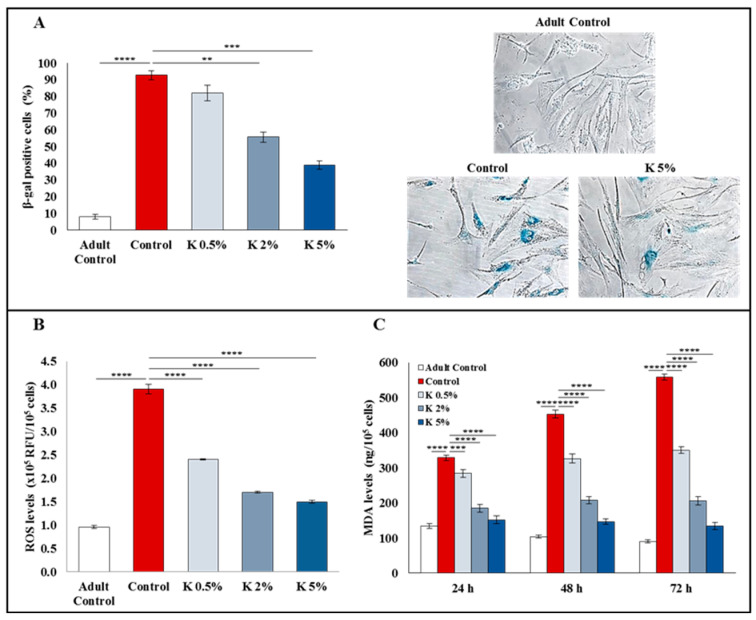
Effect of K formulation on β-galactosidase activity and oxidative stress in H_2_O_2_-induced aged HDFs. (**A**) Cellular aging was measured by β-galactosidase staining; the percentage of β-gal positive cells was determined at different K concentrations. Values are expressed as the means ± SEM of three independent experiments. Representative images of adult and aged HDFs untreated (Control) and treated for 72 h with K (5%) are also shown (20× magnification). (**B**) ROS levels were evaluated in aged HDFs, using the DCFH-DA assay after treatment. Results are relative to mean values ± SEM of two experiments performed in duplicate. (**C**) MDA levels in aged fibroblasts treated for up to 72 h as described above were assayed in cell supernatants by MDA ELISA kit. Results are relative to mean values ± SEM of three experiments performed in duplicate. For comparative analysis of groups of data, one-way or two-way ANOVA followed by Dunnett’s post hoc test was used (** *p* < 0.01, *** *p* < 0.001, **** *p* < 0.0001).

To investigate the effect of K formulation on the aging of HDFs induced by H_2_O_2_, we analyzed the β-galactosidase (β-gal) activity, known as aging biomarker. The aged cells showing high β-gal activity are stained blue. As shown in Figure 2A, less than 10% of the adult control cells were positive for β-gal activity, while more than 90% of the aged control group revealed the presence of positive β-gal cells. The exposure to K at 0.5, 2, and 5% was able to decrease the positive cell percentage (82.1% ± 4.6, 55.7% ± 3.1, and 39% ± 2.4, respectively), counteracting the H_2_O_2_-induced aging in a concentration-dependent manner, and this effect was statistically significant at 2 and 5% (Figure 2A).

The oxidative stress due to the increase in reactive oxygen species (ROS) is strictly associated with a reduced cell proliferation and subsequent cellular aging [24]. Moreover, ROS are able to promote collagen degradation, induce the accumulation of elastin, and inactivate the inhibitors of MMPs, responsible for ECM degradation [25]. The high ROS level produced by aged HDFs is responsible for the MMP expression increase and the TGF-β signaling inhibition, promoting dermal aging [26]. To investigate the antioxidant effect of K formulation in the H_2_O_2_-induced cellular aging model, we evaluated the ROS and malondialdehyde (MDA) levels. As shown in Figure 2B, the intracellular ROS levels were significantly higher in the aged controls than in the adult controls. The K treatment significantly reduced them in a concentration-dependent manner constraining H_2_O_2_-induced oxidative stress. Moreover, compared to the adult controls, the secreted MDA levels were significantly higher in the aged controls, indicating that H_2_O_2_-induced injury caused lipid peroxidation in HDFs. The K formulation treatment significantly reduced the MDA levels in a time- and concentration-dependent way (Figure 2C). These results demonstrated that the oxidative stress generated by H_2_O_2_ could be strongly counteracted by K treatment. Noteworthy, the 5% concentration was able to restore the ROS and MDA levels similar to the adult controls.

### 3.3. Effect of K Formulation on Type I Collagen Synthesis in Adult and Aged HDFs

**Figure 3:**
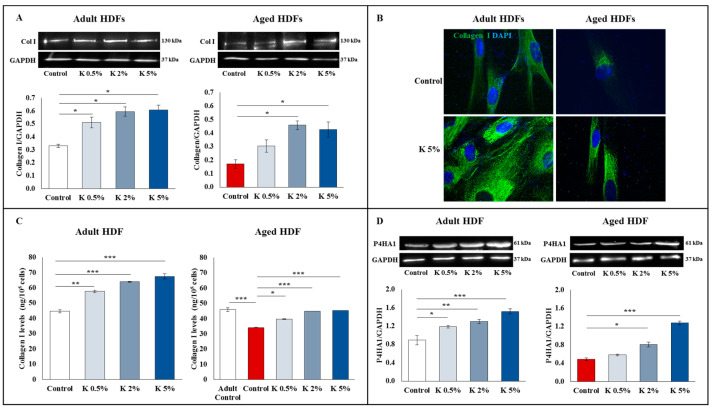
Effect of K formulation on type I collagen levels and synthesis in adult and aged HDFs. (**A**) Western blot assay for intracellular collagen I was performed on adult and aged HDFs treated with increasing concentrations of K for 72 h. The values obtained by densitometric analysis were normalized vs. GAPDH protein. Data are reported as the means ± SEM of two independent experiments. Images of representative immunoblots are shown. (**B**) Representative immunofluorescence images of adult and aged HDFs treated for 72 h with K (5%) and stained with anti-collagen I antibody (green). Nuclei were counterstained with DAPI (blue). The images are representative of three independent experiments in duplicate. All images were acquired at 100× magnification. (**C**) Collagen levels in fibroblasts treated as described above were evaluated in cell supernatants, using ELISA kit. Results are relative to mean values ± SEM of three experiments performed in duplicate. (**D**) P4HA1 levels were evaluated by Western blot analysis on adult and aged HDFs treated as described above. In all cases, the comparative analysis of data was performed using one-way ANOVA followed by Dunnett’s post hoc test (* *p* < 0.05, ** *p* < 0.01, *** *p* < 0.001).

As type I collagen predominates in the dermis and is also responsible for the tensile strength of skin tissue, we assessed if the K formulation exposure was able to influence the collagen I levels. The intracellular collagen I levels, detected by the Western blot and immunofluorescence (Figure 3A and Figure 3B, respectively), were basically lower in the aged HDFs than in the adult HDFs, as expected [27]. The Western blot analysis showed that intracellular type I collagen expression was significantly upregulated in the adult HDFs exposed to K at all tested concentrations (Figure 3A). Of note, an increase in the type I collagen levels was observed when the aged HDFs were exposed to K formulation, with significantly higher levels at 2 and 5%, compared to the aged controls. To confirm these results, we performed the immunofluorescence analysis (Figure 3B). The adult HDFs treated with the highest concentration of K displayed more intense and widespread type I collagen staining than the untreated cells. Of interest, also aged HDFs treated with 5% K showed a substantial increase in staining for collagen I compared to the relative controls. The levels of collagen secreted by the adult and aged HDFs were assayed in supernatant, using an ELISA kit (Figure 3C). Preliminary tests excluded the possibility that ELISA could detect the recombinant polypeptide α1 chain of type 1 collagen in the K formulation (not shown). In the adult HDFs, K treatment at 0.5, 2, and 5% induced a significant and dose-dependent increase in extracellular type I collagen, showing an increase percentage of approximately 26–32%, 40–46%, and 48–52%, compared to the controls, respectively. As expected, the aged HDFs secreted lower basal levels of collagen than the adult control HDFs, with a reduction of approximately 35–40%. Interestingly, also in aged HDFs, the exposure to K at 0.5, 2, and 5% induced a significant and dose-dependent increase in the extracellular collagen levels with an increase percentage of about ~15–18%, 30–35%, and 30–36%, compared to untreated cells, respectively. After K treatment, the collagen concentration in aged HDFs were restored and were not significantly different from those of the controls (Figure 3C). We also evaluated the expression of P4HA1 protein, a key player in collagen synthesis, in the adult and aged HDFs treated with increasing concentrations of K formulation for 72 h. As shown in Figure 3D, the basal levels of P4HA1 in the adult HDFs were higher than those in the aged HDFs. Interestingly, a dose-dependent increase in P4HA1 expression was observed in the adult HDFs treated with K when compared to control cells. Following K exposure, an increasing trend of the P4HA1 levels was observed in aged fibroblasts being significant at 2 and 5% (Figure 3D).

### 3.4. Effect of K Formulation on Fibroblast Contraction and Migration Activity

**Figure 4:**
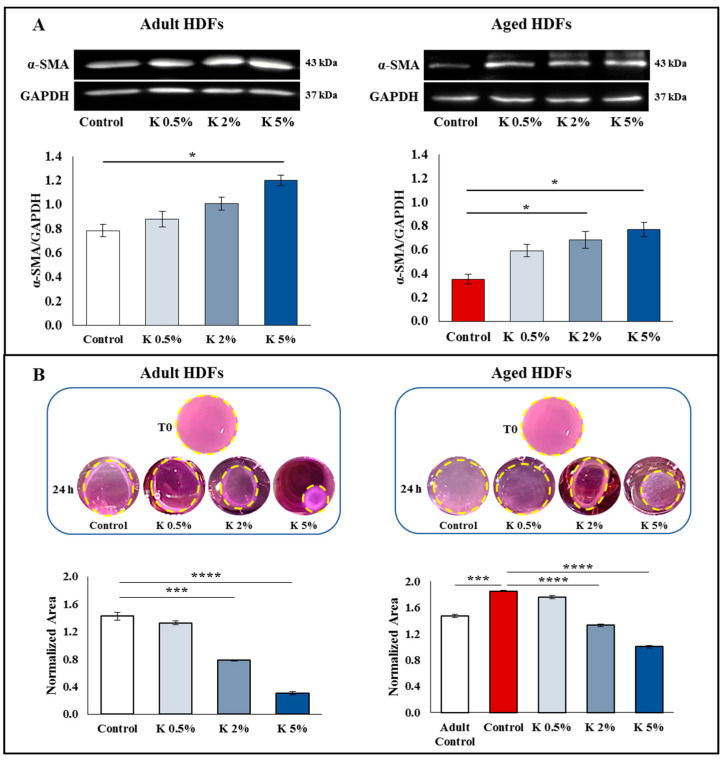
Influence of K formulation treatment on fibroblast activation. (**A**) Western blot assay for α-SMA was performed on adult and aged HDFs treated with increasing concentrations of K for 72 h. Following the densitometric analysis, the obtained values were normalized vs. GAPDH protein. Values are expressed as the means ± SEM of two independent experiments. Representative immunoblots are also shown. (**B**) Collagen gel retraction assay was performed on adult and aged HDF-populated collagen lattices following K treatment. The gel contraction was evaluated by digital camera, and the lattice area was assessed by ImageJ and normalized to pre-release area (T0). Normalized area values are reported as mean ± SEM of three independent experiments in duplicate. Representative images of lattices at T0 and 24 h after treatments are also shown. The yellow dotted circles mark the lattice’s edges. The comparative analysis of data was performed using one-way ANOVA followed by Dunnett’s post hoc test (* *p* < 0.05, *** *p* < 0.001, **** *p* < 0.0001).

As already reported [28,29], the fibroblasts basally express the α-SMA protein, which contributes to cell-generated mechanical tension, and whose expression increases in activated fibroblasts, improving their contractile activity. To verify whether the increase in collagen synthesis was associated with fibroblast contraction activity, the expression of α-SMA in the adult and aged HDFs treated with increasing concentrations of K was investigated using the Western blot method. As shown in Figure 4A, in the adult HDFs, the α-SMA protein expression enhanced in a dose-dependent way even if the upregulation was statistically significant at 5%. Of note, also in the aged HDFs, the treatment induced an increase in the α-SMA levels which was statistically significant at 2 and 5%. With the aim to evaluate the functional effect of K on the contractile machinery of the adult and aged HDFs, a 3D collagen gel contraction assay was used. The cells were seeded into a three-dimensional collagen latex, and after latex polymerization, the complete media containing increasing concentrations of K formulation were added. The results show that the K treatment at 0.5, 2, and 5% after 24 h led to a marked and concentration-dependent decrease in the collagen lattice areas with a percentage of decrease compared to the control group of approximately 5–10%, 44–47%, and 78–82%, respectively. The obtained results indicate the ability of K to improve the contractility of HDFs embedded in collagen lattices. Interestingly, the aged HDFs, which showed a lower contractility function compared to the adult controls of ~20–25%, as evidenced by the larger disc size, recovered this function with the K treatment. The polycomponent formulation was able to increase the contractility also in aged HDFs in a concentration-dependent manner (Figure 4B).

**Figure 5:**
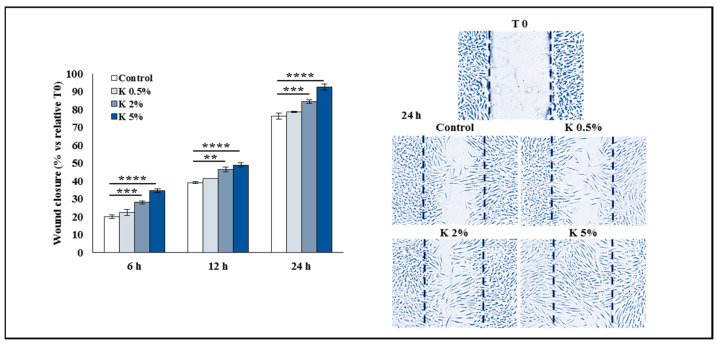
Effect of K formulation on cell migration of HDFs. The quantitative results of wound healing assay in NHDFs treated with increasing concentrations of K. The wound closure was recorded at 0, 6, 12, and 24 h after the scratch and reported as the wound closure rate (% vs. relative T0) of scratched monolayers. Showed data are the mean ± SEM of two independent experiments in duplicate. Two-way ANOVA followed by Dunnett’s post hoc test was used (** *p* < 0.01, *** *p* < 0.001, **** *p* < 0.0001) to compare the groups of data. Representative images at 24 h of NHDF monolayer re-epithelialization (the wound edges at T0 are blue dashed lines) are also shown (10× magnification).

To investigate the effect of K formulation on cell migration in the adult HDFs, the wound healing assay was performed, and the rate of scratched monolayer closure in the absence or presence of K at 0.5, 2, or 5% was evaluated by observing the repopulation of the area between the wound edges at different time points after the lesion. To quantitatively analyze the effect of K formulation on the closure of the wounded area, the images obtained by inverted light microscope were acquired at different time points after scratching and converted to percentage closure (% closure), using a mathematical model able to automatically calculate the portion of the area occupied by the cells. The analysis of an in vitro scratch assay showed that the closure percentage at 6, 12, and 24 h increased in the adult HDFs treated with K compared to the controls in a concentration-dependent way, and it was significant at 2 and 5%. Since the scratch wound assay was performed in the presence of the serum, we can conclude that K formulation was able to accelerate the wound closure, stimulating both cell migration and proliferation of the adult HDFs (Figure 5). Figure 5 shows the representative microscopic images at 24 h.

### 3.5. Effect of K Formulation on Extracellular Secretion of TGF-β1 by Adult and Aged HDFs

**Figure 6:**
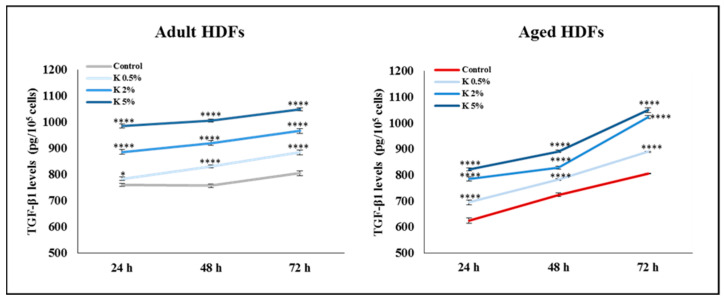
Effect of K formulation on TGF-β1 levels secreted by adult and aged HDFs. TGF-β1 levels in adult and aged fibroblasts untreated (Control) or treated for up to 72 h with K at different concentrations were measured in cell supernatants, using TGF-β1 ELISA kit. Results are from three experiments performed in duplicate (mean values ± SEM). For comparative analysis of data, two-way ANOVA followed by Dunnett’s post hoc test was performed (* *p* < 0.05; **** *p* < 0.0001).

Taken into account that TGF-β1 represents a key regulator of fibroblast proliferation, collagen production, and extracellular matrix renewal in human skin [30,31], we evaluated the extracellular secretion of TGF-β1 in the adult and aged HDFs treated for up to 72 h with increasing concentrations of K formulation. After the K exposure, the TGF-β1 levels in the supernatant of the adult HDFs enhanced in a time- and concentration-dependent way. Of note, in the aged HDFs showing the secreted basal levels of TGF-β1 lower than those in the adult cells, the K treatment induced an increase in the TGF-β1 levels at all tested concentrations (Figure 6). These results suggested that K formulation could induce the synthesis of the collagen through the TGF-β1 pathway involvement in the adult and aged HDFs.

## 4. Discussion

In the present study, we investigated the biological effects of an innovative polycomponent formulation in adult HDFs and in a H_2_O_2_-induced aging model of HDFs. Dermal fibroblasts play a crucial role in the production of ECM components and in maintaining skin homeostasis. Skin aging is characterized by reduced numbers of fibroblasts and lower levels of ECM proteins, which decrease elasticity and tonus, resulting in atrophy, wrinkling, and increased fragility of the skin [5,26]. Skin fibroblasts are largely used as cellular model for in vitro cellular aging studies. Moreover, fibroblast H_2_O_2_-induced aging is a well-known model of accelerated aging in vitro, as it mimics skin aging in vivo. Aged fibroblasts are characterized by a cell cycle arrest with long-term loss of proliferative capacity [32]. Here, we report that K treatment promoted proliferation not only in the adult cells but also in the aged HDFs, in a time- and concentration-dependent manner, and improved the changes in the cell morphology counteracting cellular aging. In addition, the K formulation reduced in the aged HDFs the percentage of cells positive for β-gal activity, an aging-associated biomarker, in a concentration-dependent manner. The increased β-galactosidase activity resulted from a rise in the number and size of lysosomes, which consequently led to an elevated lysosome content in aged cells [33].

Skin aging is closely associated with oxidative stress, a phenomenon characterized by an imbalance between reactive oxygen species (ROS) and antioxidants [34,35]. High levels of ROS oxidize cellular proteins, DNA, and lipids, inducing inflammation, oxidative damage, and aging in the dermal fibroblasts. The levels of MDA may reflect the lipid peroxidation level as well as the degree of the consequent cellular damage [36]. In particular, the MDA is able to destroy the cell membrane integrity, affect the cell structure and ion transport, and lead to dysfunction of cellular energy metabolism. We then evaluated the antioxidant activity of the K formulation against H_2_O_2_-induced oxidative stress on aged HDFs. Our findings showed that after treatment with K formulation, the H_2_O_2_-induced upregulation of the intracellular ROS and MDA levels significantly reduced in a dose-dependent manner, thus effectively exerting antioxidant effects.

One manifestation of human skin aging is losing type I collagen [26]. It is the most abundant collagen in the skin and is responsible for maintaining tissue architecture. In addition, collagen I significantly regulates many biological processes, including cell adhesion, proliferation, and differentiation. Here we show the ability of K formulation to induce a significant and dose-dependent increase in the intracellular and extracellular levels of type I collagen in adult or aged HDFs. Collagen synthesis is regulated by several post-translational modifications that are fundamental for proper stability, assembly, and secretion of triple-helical procollagen, as well as for cleavage of the N- and C-propeptides, self-assembly of collagen into fibrils, and cross-linking of the fibrils [37]. Prolyl-4-hydroxylases (P4H), an enzyme that catalyzes the hydroxylation of proline residues of procollagen, is essential for the folding of newly synthesized procollagen polypeptide chains into stable triple-helical molecules [38]. Furthermore, the P4H expression levels are associated with the rate of collagen synthesis; thus, it can be considered a “rate-limiting enzyme” in collagen production [39]. Considering the crucial role of P4H in collagen synthesis, we evaluated the ability of the polycomponent formulation to influence its expression. In the present study, the P4H levels that were basally higher in the adult HDFs compared to aged HDFs were affected by K formulation, as a significant increase in P4H was observed in both cell models as K concentrations increased. Considering that P4H is an essential determinant in initiating collagen biosynthesis, its increased expression level reflects the correct formation of a functional collagen fibril. We also show evidence that the K treatment led to a higher expression of α-SMA protein, supporting a link between the increase in collagen synthesis and activation of fibroblasts. Over the years, it has become increasingly clear that mechanical forces regulate the synthesis and remodeling of ECM proteins [40]. The contractility of dermal fibroblasts is closely related to physiological processes such as wound healing, wrinkling, angiogenesis, and inflammatory response. Notably, a reduction in collagen expression and ECM degradation can impair the attachment of dermal fibroblasts within the dermis, resulting in a change in cellular morphology with less mechanical force [41]. In our experimental conditions, the contractile activity between the adult and aged HDFs was quite different under baseline conditions; the treatment with K formulation improved the contractile activity in both models.

The TGF-β1 pathway plays an essential role in cell contractility, as it initiates the activation of fibroblasts by inducing the expression of α-SMA [41]. Moreover, TGF-β1 is a significant regulator of ECM activities, controlling the production of matrix metalloproteinases (MMPs) and serving as the primary regulator of collagen synthesis. Several in vitro studies [30,42] have shown that the ECM undergoes progressive deterioration and fragmentation during skin aging due to reduced collagen transcription resulting from the attenuated TGF-β1 pathway. Moreover, oxidative damage also impaired TGF-β1 signaling due to the elevation of ROS that, reducing the type II TGF-β receptor (TβRII) and SMAD3 protein levels, decrease collagen synthesis [43,44]. Indeed, aged HDFs showing a significantly lowered TGF-β1 expression express less collagen than young fibroblasts. Our results show the ability of K formulation to induce a dose- and time-dependent increase in the TGF-β1 secretion relative to the controls in both models. Our findings support the notion that this polycomponent formulation could efficiently activate TGF-β1 signaling in fibroblasts, significantly synthesizing collagen I.

To the best of our knowledge, this is the first study showing in vitro the biological effects of a polycomponent formulation of non-crosslinked HMW-HA, recombinant polypeptide of collagen-1 chain, and CMC. Moreover, our results add to the growing literature indicating that in vitro cellular models may be viable tools for testing the effect of medical devices without resorting to an animal study. Indeed, according to the European Directive 63/2010/EU, in vitro methods could significantly contribute to limiting the use of animal models to evaluate the biological response of medical devices.

An overall interpretation of our results is that the combination of these bioactive components, being able to improve the biological activities in adult fibroblasts, as well as counteract H_2_O_2_-induced fibroblast aging, could be used to maintain skin homeostasis, accelerate post-wounding healing, and treat critical age-related skin signs. Additional research will be needed to deepen further understanding of the involvement of signaling pathways underlying the polycomponent formulation’s antiaging and antioxidant effects and evaluate its ability to prevent fibroblast aging.

## Data Availability

The datasets generated and analyzed during the current study are available from the corresponding authors upon reasonable request.
